# A peptide-loaded dendritic cell based cytotoxic T-lymphocyte (CTL) vaccination strategy using peptides that span SIV Tat, Rev, and Env overlapping reading frames

**DOI:** 10.1186/1742-4690-3-1

**Published:** 2006-01-06

**Authors:** Zachary Klase, Michael J Donio, Andrew Blauvelt, Preston A Marx, Kuan-Teh Jeang, Stephen M Smith

**Affiliations:** 1 Department of Infectious Diseases, Saint Michael's Medical Center, Newark, New Jersey, USA; 2 Department of Preventive Medicine and Community Health, New Jersey Medical School, Newark, New Jersey, USA; 3 Department of Dermatology, Oregon Health & Science University, Portland, Oregon, USA; 4 Department of Molecular Microbiology and Immunology, Oregon Health & Science University, Portland, Oregon, USA; 5 Dermatology Service, VA Medical Center, Portland, Oregon, USA; 6 Tulane National Primate Research Center, Tulane University Health Sciences Center, Department of Tropical Medicine, Covington, Louisiana, USA; 7 Molecular Virology Section, Laboratory of Molecular Medicine, NIAID, NIH, Bethesda, Maryland, USA

## Abstract

CTL based vaccine strategies in the macaque model of AIDS have shown promise in slowing the progression to disease. However, rapid CTL escape viruses can emerge rendering such vaccination useless. We hypothesized that such escape is made more difficult if the immunizing CTL epitope falls within a region of the virus that has a high density of overlapping reading frames which encode several viral proteins. To test this hypothesis, we immunized macaques using a peptide-loaded dendritic cell approach employing epitopes in the second coding exon of SIV Tat which spans reading frames for both Env and Rev. We report here that autologous dendritic cells, loaded with SIV peptides from Tat, Rev, and Env, induced a distinct cellular immune response measurable *ex vivo*. However, conclusive *in vivo *control of a challenge inoculation of SIVmac239 was not observed suggesting that CTL epitopes within densely overlapping reading frames are also subject to escape mutations.

## Background

Several recent HIV vaccine strategies have focused on the induction of potent cellular immune responses [1]. Experiments in the macaque model of HIV infection have shown that a strong cytotoxic T-cell lymphocyte (CTL) response against viral proteins can prevent disease, although such a response cannot prevent infection. Unfortunately, viruses which escape CTL-surveillance frequently occur in animals, and such escaped viruses can then engender disease [2].

Most vaccines have used whole viral proteins, delivered in a variety of ways, as immunogens. While some of these proteins in the context of particular major histocompatibility (MHC) antigen alleles show immunodominant epitopes in macaques [3,4], a general strategy is to induce broad CTL responses against many different epitopes. Several CTL-eliciting epitopes can be present in a given protein. To date, HIV/SIV has been able to generate escape mutations within most, if not all, epitopes used to elicit CTL-responses. Many such mutant viruses can replicate to high levels and cause disease *in vivo *suggesting that these mutated viruses do not have significantly reduced viral fitness [5]. However, the true functional content of the many CTL-eliciting epitopes used for vaccination has not been clearly defined.

Since CTL based vaccines reduce, but do not eliminate replication, it is expected that they will select for the emergence of escape viral mutants. For a CTL based vaccine to be durably effective, ideally, the target epitope(s) must be critical for function and be constrained such that any change in epitope sequence results in a significant deficit in the replicative fitness of the virus. Thus, in an operational definition, an "immutable" CTL epitope is one which may mutate in response to immune selection, but such mutations are transient and may never be observed because of their significantly deleterious effect on viral fitness.

In a recent study, we explored the concept of such an immutable epitope [6]. We infected macaques with an engineered version of SIVmac239 (i.e. SIVtat1ex) which can only express the first coding exon of SIV Tat due to artificially inserted premature stop codons that prevented expression of the second coding exon of Tat. SIVtat1ex virus replicated well in the early phase, but much less well than wild type (i.e. SIVtat2ex) in the chronic phase of infection. In three macaques, SIVtat1ex "reverted" and opened up the stop codons that obstructed expression of the second coding exon of Tat (i.e. SIVtat1ex became SIVtat2ex). In two of these three animals, this change in Tat expression (i.e. expression of full length two-exon Tat instead of the original one-exon Tat) correlated with increased viral load and more rapid CD4^+ ^T-cell depletion. In the third animal, the viral load initially increased, but then returned to low levels. Further investigation revealed that this third animal, although originally infected with SIVtat1ex, transiently had the emergence of a SIVtat2ex virus which surprisingly reverted quickly back to the less fit SIVtat1ex form. This third macaque has maintained low viral load and high CD4^+ ^T-cell count. Immunologic studies demonstrated that this animal had a strong cellular response directed to the second coding exon of SIV Tat. Provocatively, after 4 years of infection, this animal continued to maintain the low-fitness SIVtat1ex virus with no evidence for the ability of the more fit SIVtat2ex to emerge by correcting the stop codons which prevent the expression of the second coding exon of Tat. Our interpretation of this scenario in the context of our operational definition of an "immutable CTL epitope" is that SIVtat2ex is a transitional "escape" virus of SIVtat1ex; and that in certain settings SIVtat1ex virus cannot durably transit to its more fit SIVtat2ex form because the host maintains a potent CTL selection targeted against an epitope within the second coding exon of Tat.

**Figure 1:**
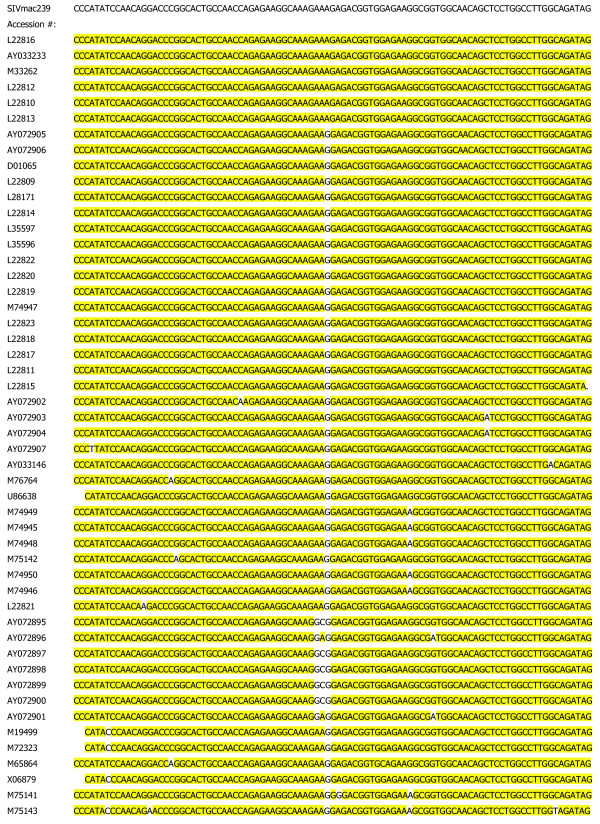
Nucleic acid alignment of the second exon of Tat for all SIVmac strains relative to SIVmac239. Highlighted residues are identical to that of SIVmac239.

An inference from our above interpretation is that the second exon of Tat is functionally important to viral fitness, and mutation(s) within this region is detrimental to viral replication *in vivo*. Moreover, because the Rev and Env proteins are expressed from reading frames that overlap the second coding exon of Tat, we believe that such overlap might be an additional reason for the "immutability" of this region. Compatible with this notion is the fact that viral sequences in this region are remarkably well conserved (Figure 1). Because mutations that affect the coding region for Tat can also unintentionally perturb the coding sequences of Rev and Env, one issue which we wished to investigate is whether the protein coding density of a region might constrain HIV-1 against developing mutations.

The above hypothesis posits that mutations in a CTL-epitope(s) embedded within a portion of SIV that codes three overlapping proteins, Tat, Rev and Env, might be difficult. The notion is that such CTL-epitopes might be "immutable" because "escape" changes in their sequences could alter Tat, Rev, or Env function (singularly or multiply) in ways that produce less-fit progeny viruses *in vivo*. Peptide loaded dendritic cells have been used in cancer immunotherapy and in viral vaccine efforts to induce a cellular response against specific epitopes [7,8]. To test our hypothesis that triply over-lapping reading frames potentially restrict CTL-escapes, we immunized macaques with autologous dendritic cells, loaded with peptides from an SIV region with overlapping coding capacity for Tat, Rev, and Env. Here, we report findings when we challenged immunized animals with a pathogenic SIVmac239 virus.

## Results

### Peptide-loaded dendritic cells elicited strong IFN-γ T-cell responses

To assess the effectiveness of the dendritic cell culture protocol, we performed flow cytometry for the MDDC phenotype. After 8 days in culture, cells were stained for HLA-DR and CD83. Immature dendritic cells express relatively low levels of HLA-DR and are CD83 negative, whereas mature dendritic cells express higher levels of HLA-DR and are CD83 positive. Flow cytometry revealed that greater than 80% of the cultured MDDC possessed the mature phenotype (data not shown).

Previously, others have shown that surface MHC molecules on dendritic cells bind soluble peptides or portions of them during tissue culture [11]. After injection of peptide loaded MDDC into animal hosts, MDDC can present these peptides to T-cells and can induce strong cellular responses against the peptides. To stimulate specific cellular responses, each animal in the experimental group was injected with autologous mature MDDC, which had been cultured in the presence of peptides from the overlapping regions of Tat, Rev, and Env (Table 1). The SIV peptides used have identical sequences to those encoded by the challenge virus, SIVmac 239. Four animals, AT56, AT57, AV89 and BA20, were selected for the experimental group. The remaining two, H405 and T687, were assigned to the control group. Control animals were injected with autologous mature MDDC, which were cultured in the absence of SIV peptides. The MDDC were injected into an inguinal lymph node, which was located by palpation. Each animal received 6 vaccinations over 83 days.

**Table 1:** Amino acid sequence of peptides from Tat, Rev, and Env used in the vaccine. (*Peptide sequences are identical to those of challenge virus*.)

**Tat**		**Rev**		**Env**	
5429	TPKKAKANTSSASNK	6089	RLRLIHLLHQTNPYP	6708	YRPVFSSPPSYFQQT
5430	AKANTSSASNKPISN	6090	IHLLHQTNPYPTGPG	6709	FSSPPSYFQQTHIQQ
5431	TSSASNKPISNRTRH	6091	HQTNPYPTGPGTANQ	6710	PSYFQQTHIQQDPAL
5432	SNKPISNRTRHCQPE	6092	PYPTGPGTANQRRQR	6711	QQTHIQQDPALPTRE
5433	ISNRTRHCQPEKAKK	6093	GPGTANQRRQRKRRW	6712	IQQDPALPTREGKER
5434	TRHCQPEKAKKETVE	6094	ANQRRQRKRRWRRRW	6713	PALPTREGKERDGGE
5435	QPEKAKKETVEKAVA	6095	RQRKRRWRRRWQQLL	6714	TREGKERDGGEGGGN
5436	AKKETVEKAVATAPG	6096	RRWRRRWQQLLALAD	6715	KERDGGEGGGNSSWP
5437	TVEKAVATAPGLGR	6097	RRWQQLLALADRIYS	6716	GGEGGGNSSWPWQIE
		6098	QLLALADRIYSFPDP	6717	GGNSSWPWQIEYIHF
		6099	LADRIYSFPDPPTDT	6718	SWPWQIEYIHFLIRQ
				6719	QIEYIHFLIRQLIRL

* Note: Each peptide is identified by its AIDS Reagent catalog number.

**Figure 2:**
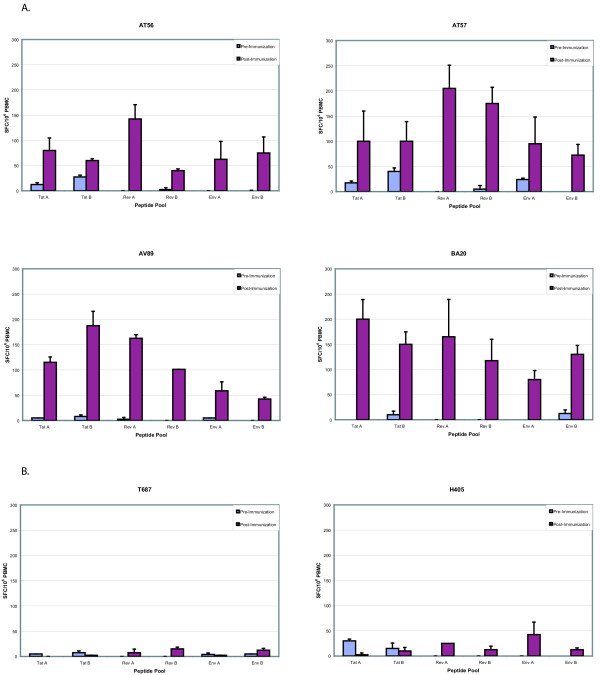
IFN-γ T-cell responses against the overlapping epitopes of Tat, Rev, and Env. Using an ELISpot assay, we measured IFN-γ T-cell responses against the peptides used in the vaccination protocol. The vaccinated animals (Panel A), AT56, AT57, AV89, and BA20, each developed strong to moderate responses against every peptide pool tested at a least one time point, except AT56 against Rev pool B. The control animals (Panel B), H405 and T687, did not demonstrate any significant activity throughout the study. The activity levels against the peptide pools, Tat A, Tat B, Rev A, Rev B, Env A, and Env B, are shown for each animal in spot forming cells (SFC) per 10^6 ^PBMC. Data are shown from pre-immunization and post-immunization assays. The average numbers of SFC per PBMC and the standard deviations (error bars) were determined from duplicate wells. Responses greater than 50 SFC/10^6 ^PBMC were considered positive.

The MDDC vaccine approach was chosen to generate cytotoxic T-cell lymphocytes specific to these SIV peptides. The SIV specific response was measured by an interferon gamma (IFN-γ) ELISpot assay. For *in vitro *culturing purposes, the peptides were arbitrarily divided into six pools (see Methods section): Tat A, Tat B, Rev A, Rev B, Env A, and Env B. Experimental animals (AT56, AT57, AV89, BA20) developed strong IFN-γ T-cell responses to all vaccinated peptide pools over the course of the six vaccinations (Figure 2A). Responses to each peptide pool grew from baseline to greater than 50 SFC/10^6 ^PBMC for at least one time point in all animals except AT56, which did not develop a response to the Rev B pool. Control animals (H405, T687) consistently had responses less than 50 SFC/10^6 ^PBMC to each pool (Figure 2B).

### Lack of control with viral amino acid changes when SIVmac239 challenge virus was used to infect peptide immunized macaques

**Figure 3:**
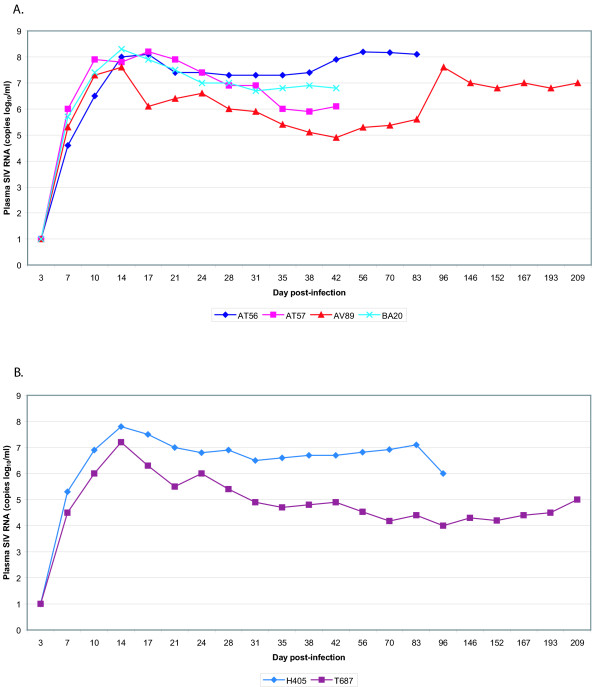
SIVmac239 plasma viremia over time. Plasma samples were measured from the corresponding time points for SIV RNA concentration via the bDNA assay. The data from the vaccinated animals (AT56, AT57, AV89, and BA20) are shown in Panel A, while those from the controls (H405 and T687) are shown in Panel B.

Six days following the final vaccination (Day 89 of the study), each animal was intravenously challenged with 50 infectious units of SIVmac239. Plasma viremia occurred in each animal and reached a peak by Day 14 post-infection. There were no discernible differences between the viral loads of the experimental animals and the control animals (Figure 3). The analysis of the study was complicated by two animals, BA20 and AT57, becoming ill very shortly after SIV infection. BA20 began losing weight towards the end of the vaccination period. BA20's weight fell from 9.25 kg to 7.45 kg by day 99 (day 10 post challenge). Blood work revealed an elevated white cell count. The animal lost weight progressively. AT57 began losing weight around Day 14 post-challenge, suffered a 23% drop in hematocrit levels, and was found to have a firm mass in the abdomen. Both animals became clinically ill and were culled 42 days post-infection. Necropsy showed that AT57 died of metastatic endometrial cancer. Necropsy and subsequent histology of BA20 determined the cause of death to be gastroenterocolitis. In both animals, it is unlikely that SIV infection contributed to their morbidity.

**Figure 4:**
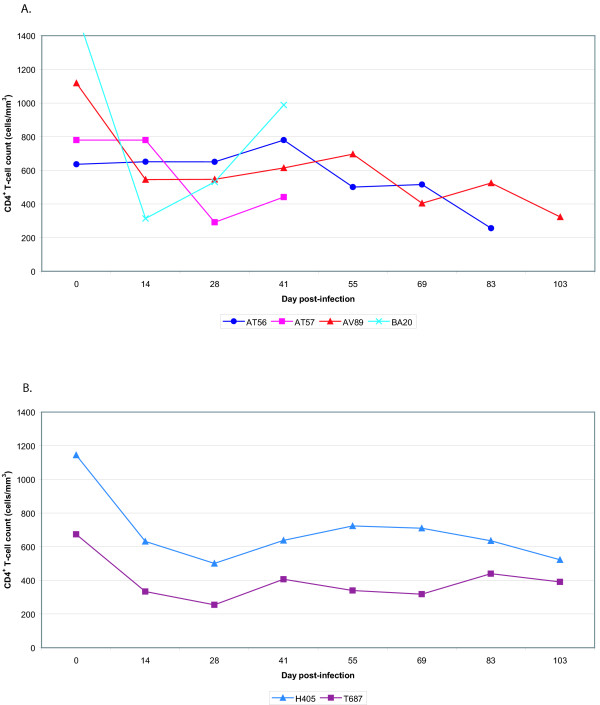
CD4^+ ^T-cell counts. Peripheral blood CD4^+ ^T-cell counts were longitudinally determined by flow cytometry. The data from the vaccinated animals (AT56, AT57, AV89, and BA20) are shown in Panel A, while those from the controls (H405 and T687) are shown in Panel B.

**Figure 5:**
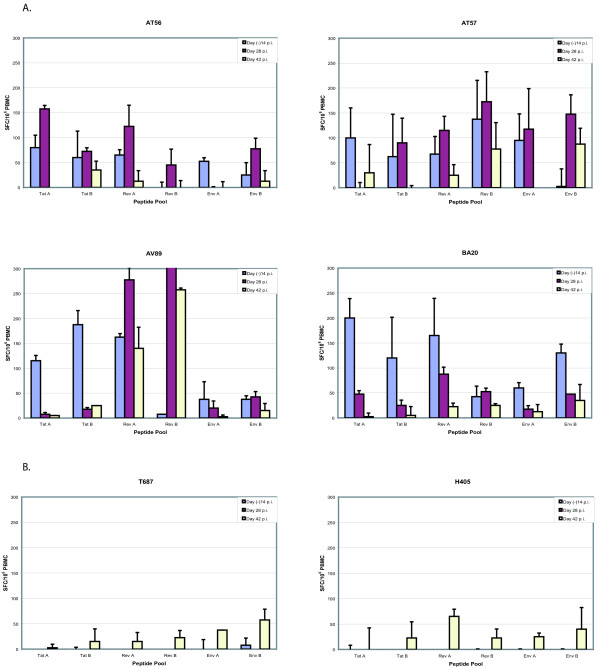
IFN-γ T-cell responses against Tat, Rev, and Env after SIV infection. IFN-γ T-cell responses of the vaccinated animals were again measured by a IFN-γ ELISpot assay on PBMC from Days 28 and 42 post-infection. The activity levels against the peptide pools, Tat A, Tat B, Rev A, Rev B, Env A, and Env B, are shown for each animal in SFC per 10^6 ^PBMC at Day 74 (14 days prior to infection), Day 28 post-infection (p.i.), and Day 42 p.i. In most instances, the activity decreased significantly by Day 42 p.i. AV89's strong response against Rev (430 SFC/10^6 ^PBMC) was not shown, so that the y-axis maximum would be the same for each graph.

The CD4^+ ^T-cell counts in most animals declined over the first few weeks post-infection (Figure 4). BA20 began a 4-week rise in CD4^+ ^T-cell count before being culled. Remaining animals maintained a CD4^+ ^T-cell count between 400 and 600. AT56 and H405 began to show symptoms of simian AIDS and were culled approximately 3 months after infection. Plasma viral loads rose rapidly in all animals to a peak level at day 14 before declining to set point. AT56 and H405 maintained relatively high viral loads, greater than 10^7 ^copies/ml until being culled. Other animals maintained lower viral loads, from 10^5 ^to 10^7 ^copies/ml. AV89 and T687 remained healthy for over 1.5 years after infection. The SIV cellular activities against the Tat, Rev, and Env peptides were measured at 28 and 42 days post-challenge. In three of the four animals, the cellular responses dramatically decreased by day 42 of SIV infection (Figure 5). No significant SIV specific IFN-γ T-cell activity developed in either control animal after SIV infection.

**Figure 6:**
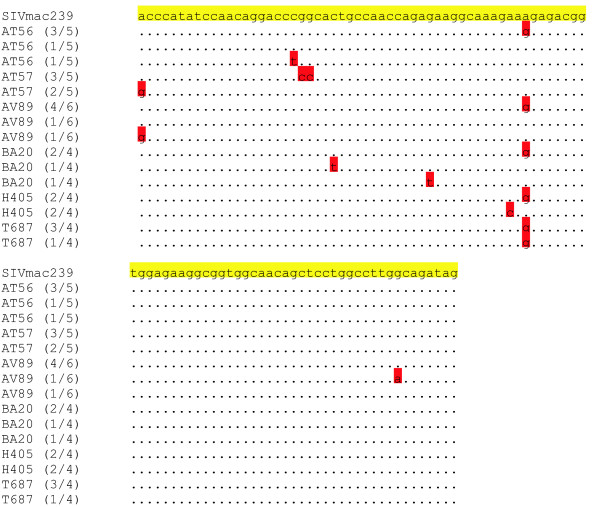
Viral sequences from Day 28 are compared to SIVmac239, the challenge virus. Viral RNA was extracted from each animal's plasma on Day 28. After RT-PCR, cDNAs were cloned into a plasmid. Individual clones were then isolated and sequenced. In parentheses, the numerator indicates the number of clones with a given sequence and the denominator shows the total number of clones sequenced. Mutations are highlighted in red.

**Figure 7:**
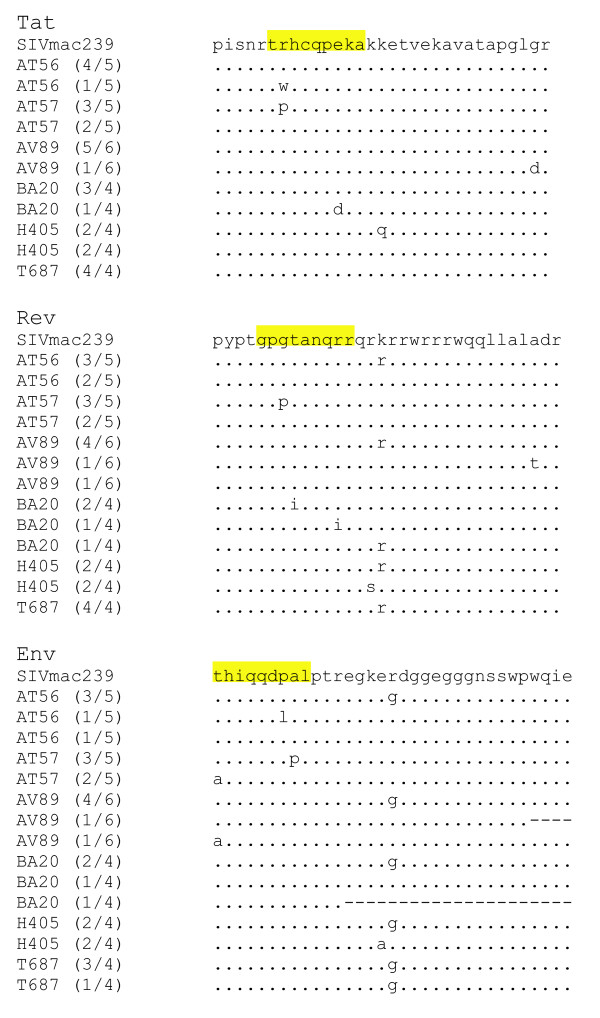
Encoded amino acids from Day 28 viruses in Tat, Rev, and Env from the overlapping regions used in the peptide vaccination. Proposed CTL epitopes are highlighted in yellow.

Changes in nucleic acid sequences of virus isolates were determined longitudinally over the course of infection. By day 28 post infection five of the animals (AT56, AV89, BA20, H405 and T687) had developed an A to G mutation at bp 8854 affecting Rev and Env, which quickly became the dominant species and that corresponded to a previously identified sub-optimal nucleotide in the SIVMac239 molecular clone [12]. Several mutations in each of the three reading frames were seen at Day 28 (Figures 6 &7). We interpret the emergence of these amino acid changes in the face of a lack of *in vivo *control of challenge virus to mean that CTL-responses in *a priori *coding-frame dense portions of the SIV genome are not sufficient to restrict the development of viral escape mutants.

## Discussion

In this study, we show that autologous dendritic cells, loaded with exogenous SIV peptides, can successfully induce cellular immune responses. These responses were moderate to strong, and, in general, increased with repeated immunization (data not shown). However, the vaccinated macaques seem not to effectively control the replication of a challenge virus, and inoculated animals developed viral loads similar to those of the control animals (Fig. 3). Curiously, rather than increasing after infection with SIV, the IFN-γ T-cell responses against the vaccine peptides decreased in three of the four vaccinated animals (Fig. 5). These findings are perplexing; and the unexpected early, study-unrelated demise of two experimental animals also contributed difficulties to a conclusive interpretation.

How could one explain the above findings? We note with interest the sequencing results on virus samples isolated from infected animals on day 28 after challenge (Figs. 6 &7). A close examination of the Tat sequences in Table 4 instructively suggests that the challenge virus appears to have commenced sequence changes, possibly evolving as a result of the host's CTL. Thus, if a dominant CTL epitope in SIV Tat were to span the **trhcqpeka **sequence, then in three of the four (75%) experimental animals (AT56, AT57, and BA20) viruses have initiated evasive amino acid mutations. Correspondingly, in the Rev sequence, if a major CTL epitope resided in **gpgtanqrr**, then viruses in AT57 and BA20 (50% of the experimental animals) would have started to change. A similar case could be made for Env. If the dominant epitope here is hypothesized as **thiqqdpal**, then three of the four viruses in vaccinated animals (AT56, AT57, and AV89; i.e. 75% of the experimental population) have changed by day 28. It remains to be established whether our hypothesized epitopes are truly the dominant *in vivo *SIV moieties. However the observation that the originally detected CTL responses faded quickly after virus challenge is compatible with these being relevant epitopes. Viral escape changes in these epitopes are expected to result in failure to re-stimulate the original CTL and would be consistent with the waning CTL profiles in Figure 5.

We note a sobering take home lesson from our study. Our data appear to tell us that one of our *a priori *facile assumptions is probably incorrect. We had assumed that just because a region of the virus is ORF dense that such region would be functionally constrained and difficult to mutate. The empirical results do not support that assumption. For example, the "k" in the middle of the Rev sequence seems to be easily changeable; as is the "r" in the middle of Env (Fig. 7). Neither is a result of immune selection, since viruses in the control animals also had these changes. Add to these mutations the additional changes seen in the viruses in vaccinated macaques, then the reality emerges that three densely over-lapping reading frames in a small region does not seem to greatly constrain virus mutability. Currently, we cannot formally conclude whether the viral changes in the vaccinated animals resulted in reduced fitness (however slight). Nonetheless, the *in vivo *viral replication profiles (Fig. 3) would seem to argue against this possibility.

We do want to point out several technical shortcomings to our study. First, our study group size was small and was unexpectedly confounded by the need to euthanize two vaccinated animals shortly after SIV challenge. One macaque became ill from an unrelated neoplasm, and the second developed severe enterocolitis, also believed to be unrelated to SIV, since the disease preceded SIV infection. This unanticipated happenstance reduced our vaccinated group from 4 to 2 animals and prevented a meaningful longer chronological follow up of viral sequence changes. Second, our CTL epitope interpretations are complicated by the current poor understanding of the MHC-context for rhesus macaques [13]. Since CTL-responses are MHC dependent, a fuller understanding of macaque MHC would be helpful to design and study better CTL-vaccination in monkeys. Finally, our dose of challenge virus may be too high to see obvious protection. There could be a lower dose at which a CTL response would rapidly control the virus preventing the virus from replicating enough rounds to generate an escape variant. The above caveats aside, our current results suggest that a CTL vaccine based on the Tat, Rev, Env ORF-dense region of SIV is largely insufficient (under the currently utilized challenge condition) to control virus replication. Whether protocols of immunization with Tat, Rev and Env different from those currently employed here can exert control over virusreplication remain to be investigated. Currently, we also cannot distinguish between whether the immune responses observed in our animals were qualitatively ineffective at controlling infection or if higher quantitative immune responses were induced such could, in fact, control viral infection.

## Methods

### Animals

Six colony-bred rhesus macaques (*Macca mulatta*) were obtained from the Tulane National Primate Research Center (TNPRC) (Covington, LA). The six adult animals weighed between 6.15 to 10.25 kg, and were all seronegative for SIV. All aspects of this study were approved by the Tulane National Primate Research Center Institutional Animal Care and Use Committee.

### Peptides

The SIV peptides were obtained from the NIH AIDS Reagent Program (Rockville, MD). Each was fifteen amino acids in length, and overlapped adjacent peptides by eleven residues. Nine peptides were selected which completely overlapped the second exon of SIVMac239 tat 2^nd ^exon (amino acids 98–130). Eleven Rev and twelve Env peptides were selected because their coding sequences completely or partially overlapped Tat's second exon (Table 1). The peptides from each protein were arbitrarily divided into two pools, A & B. Each pool contained 4–6 peptides. For instance, Tat pool A contained the first five peptides listed in Table 2 and Tat pool B contained the remaining four. The peptides for a given pool were dissolved together in water or DMSO at 5 mg/ml of each peptide. Each peptide exactly matched the encoded, cognate peptide of the challenge virus, SIVmac239.

### Cell culture/vaccine generation

Primary blood mononuclear cells (PBMC) were separated from heparin treated rhesus macaque blood by centrifugation over Ficoll (Greiner Inc, Longwood, FL), washed, and cryo-preserved until needed for generation of dendritic cells. For each vaccination, 2.5 × 10^7 ^PBMC per animal were thawed, washed in PBS, plated across a 6-well costar plate in DMEM with 10% FBS, and placed in a 37°C/5% CO_2 _to allow monocyte adherence. After three hours, the media and non-adherent cells were aspirated, and the plates washed twice with PBS. Media was replaced with DC media (RPMI with 10% FBS, 50 ng/ml GMCSF (R&D Systems, Minneapolis, MN) and 10 ng/ml IL-4 (R&D Systems). Cells were allowed to differentiate for 4 days. On day 4 immature dendritic cells were aspirated from the plate and washed. Cells were resuspended in 5 ml DC maturation media (RPMI 10% FBS, 50 ng/ml GM-CSF, 10 ng/ml IL-4, 20 ng/ml TNF-α, 20 ng/ml IL-6, and 20 ng/ml IL-1β (R&D Systems)) in a T25 flask. Dendritic cells from experimental animals (AT56, AT57, AV89 and BA20) received 5 μg/ml each of the Tat, Rev and Env peptides. After four additional days in culture, mature monocyte-derived dendritic cells (MDDC) were removed from culture flasks, brought to 10 ml with DC maturation media, counted, and transferred to a 15 ml conical tube for shipment to TNPRC. MDDC cultures were analyzed by flow cytometry on a FACS Calibur (BD Biosciences, Franklin Lakes, NJ).

### Vaccination

Six vaccinations were scheduled, at two-week intervals. Vaccination number five was delayed for two weeks, thus pushing back vaccinations number five and six. For each time point MDDC were generated as above and shipped overnight at room temperature to TNPRC. After centrifugation, 1 – 2 × 10^6 ^mature autologous dendritic cells were resuspended in 0.2 ml PBS and injected into a femoral lymph node in each animal. Experimental animals (AT56, AT57, AV89, BA20) received MDDC generated in the presence of Tat, Rev and Env peptides. Control animals (H405, T687) were cultured in the absence of peptides.

### Challenge

The challenge virus, a generous gift of David Watkins, University of Wisconsin, was SIVMac239(open) produced from transfected DNA and expanded in CEMx174 cells. Viral stock was diluted to 50 TCID_50_/ml in DMEM and 1 ml was administered intravenously to all animals six days after the final vaccination.

### ELISpot

IFN-γ ELISpot assay (adapted from Amara et al[9]) was performed on fresh PBMCs isolated from heparin treated blood. In brief, Multiscreen HA plates (Millipore, Billerica, MA) were coated with mouse anti-human IFN-γ (Pharmingen) and incubated overnight at 4°C, washed with PBS 0.1% Tween, loaded with 2 × 10^5 ^PBMC per well, and 5 μg/ml of the peptide pool, in duplicate. Plates were incubated at 37°C in a CO_2 _incubator for 48 hours, washed, treated with a biotinylated anti-human IFN-γ (MabTech), and then developed using streptavidin-HRP (Pierce) and Stable DAB (Research Genetics). Spot forming cells (SFC) per million PBMC were determined by subtracting the average background value for each animal from the average of the duplicate wells and multiplying by five.

### Viral load and CD4^+ ^T-cell counts

Plasma samples were separated and stored at -80°C until assayed. Plasma viral loads were quantified by the Bayer SIV bDNA assay (Bayer Reference Testing Laboratory, Emeryville, CA)[10]. Peripheral blood CD4^+ ^T-cell concentrations were quantified using standard techniques, as previously described[6].

### Sequence analysis

At two-week intervals following challenge plasma was obtained from animals for viral sequence analysis. RNA was extracted from plasma samples by Qiagen RNA Isolation kit. The Tat 2^nd ^exon was amplified by reverse transcription followed by two rounds of nested PCR. Primers used were; 1^st ^round forward – TGAGACTTGGCAAGAGTGG, 1^st ^round reverse – GGACTTCTCGAATCCTCTGTAG, 2^nd ^round forward – GGTATAGGCCAGTGTTCTCT, 2^nd ^round reverse – TATCAGTTGGCGGATCAGGA. Second round PCR fragment was 173 bp in length and corresponded to SIVMac239 base pairs 8762 to 8934 (GenBank accession # M33262) Fragments amplified by PCR were TA-cloned by topoisomerase into pCR2.1Topo (Invitrogen). Sequencing was performed using M13-reverse primer.
